# Book Review: Research ethics in the life sciences

**DOI:** 10.3389/adar.2024.12793

**Published:** 2024-04-22

**Authors:** Stephanie J. Bird

**Affiliations:** Retired, Wrentham, MA, United States

**Keywords:** research ethics, research integrity, responsible conduct of research, RCR, RCR education, collegiality

**A Book Review on**

**Research ethics in the life sciences, Third Edition**

by Kuhar Michael J. 2022, 151 pages, Paperback ISBN: 979-8-4139-6760-7

## Introduction

The research community has come to recognize that neither understanding scientific principles, nor facility with research techniques, separately or together, are sufficient for professional success. Also necessary is competence with regard to a wide range of elements that, as a group, compose what has become the field of research ethics (also known as the responsible conduct and reporting of research, or RCR for short^1^). These elements reflect both the expectations, and the conscious and unconscious assumptions that colleagues and collaborators, and society, have of each other regarding professional behavior and the conduct of research.

The aspects of research that are explicitly considered in research ethics are more or less common to all fields of research and include (but are not limited to) such topics as data management, publication and authorship practices, recognizing and addressing conflicts of interest, dealing with research misconduct (generally identified as fabrication or falsification of data, and plagiarism), the care and use of research subjects whether human or not, and the responsibilities of mentors. The specific details of accepted practice may vary depending on the field of research. Taking authorship as an example, in some fields authors are listed alphabetically while in others authors are ranked in descending order of the extent or importance of their contribution to the published work.

Whatever the details of the responsible conduct of research in a particular discipline, it is essential that this information be communicated effectively to trainees. Leaving students to learn and adopt professional standards by interpreting the observed behavior of more senior professionals is inadequate and unreasonable, in part because, often, neither practices nor the policies and assumptions on which they are based are articulated or explained. Further, not all professionals are aware of, or would agree on, disciplinary conventions and best practices. In some cases, rules may be provided in regulations or codes of ethics available online; however, explicit, interactive discussion with senior professionals is more effective and meaningful. Identification of specific practices and their underlying principles, goals, and objectives, in combination with an in-depth discussion of illustrative cases and examples, is significantly more helpful to trainees. Further, this information is more likely to be understood and adopted by students and trainees when it is identified as essential to professional development, and presented by faculty who are well-regarded by colleagues, in an open and interactive manner that clarifies the range of accepted practices, as well as problematic and unacceptable practices.

## Summary of the book

As a well-known, successful, and highly-respected senior researcher, Michael J. Kuhar is an ideal author on this topic. His contribution to discussion of the responsible conduct and reporting of research, *Research Ethics in the Life Sciences*, is written expressly for students and trainees new to research in the life sciences. It is an introduction with brief chapters complemented by a variety of online resources. The focus on trainees is apparent in the initial chapters, the first on mentor-trainee relationships and the second on the basic assumptions and foundations of the research process. These are followed by chapters on the usual RCR topics: animal and human experimentation; data acquisition, management, sharing and ownership; authorship; peer review; research misconduct; and conflicts of interest and commitment. It should be noted that, in addition to this array of RCR topics, Kuhar adds chapters on stem cells and gene editing (an ethically rich topic not usually included in RCR education), and collegiality (in a chapter entitled “Ethical Behavior Among Colleagues”). The latter is an area to which Kuhar has made significant contributions. In each chapter, the text is accompanied by short cases on which Kuhar provides some commentary.

## Evaluation of the book’s content

*Research Ethics in the Life Sciences* is a good if somewhat superficial overview. It is a good match to its audience and likely to be a conversation starter. A major strength of this work is that, unlike many of the other texts used for introducing and teaching new researchers about the ethics of research practice, it provides the perspective of a senior and very successful researcher who understands and presents the established views, perspectives and generally unarticulated assumptions and expectations that have been passed down from one generation to the next. This can provide a helpful orientation for a thoughtful, young researcher entering the field. Further, also unlike other similar texts, Kuhar’s *Research Ethics* explicitly highlights the role and importance of collegiality as an aspect of collaborative research. Overall, this work is a good introduction for those new to the topic of research ethics. It will doubtless trigger conversations as researchers consider the issues, some of which are highlighted in the cases.

The chapter on data acquisition and management (in combination with the chapter on premises, rigor and reproducibility) is especially informative with a good deal of practical advice. On the other hand, given the specific target audience, the chapters on authorship and mentorship are somewhat disappointing. Because recognition through authorship generally serves as the basis for hiring, promotion and funding throughout one’s career as a researcher, it is crucial that, early on, trainees have reasonable expectations regarding authorship, and as clear an understanding as possible of the range of accepted practices, and the prevalence of issues and assumptions that underlie various questionable authorship practices. This should include approaches for addressing authorship disputes as well as strategies for discerning the particular policies of individual research supervisors and senior researchers with regard to the nature and extent of contributions that merit authorship credit.

The chapter on mentorship stresses the responsibility of trainees who, at least initially, are disadvantaged by limited experience in research and even less experience in negotiating the challenging terrain of academia and the politics of a workplace setting. Emphasis on the responsibility of trainees creates a research climate that is less than nurturing of those who are interested and capable of pursuing careers in science, and instead, is indirectly focused on counterproductive weeding out of trainees who struggle with deciphering unspoken expectations and assumptions. At the same time, this chapter emphasizes the independence and the role responsibility of the research supervisor or head of the research group. This is an important “Take Home” message for research trainees. Ultimately, it is the head of the research team who sets the tone of the research environment both in the individual workplace setting, and in society as a whole, for better or for worse. In so doing, it is the head of the research group who is responsible for what the next generations of researchers learn, both explicitly, and implicitly through the “hidden” curriculum that is the day-to-day workplace experience.

Kuhar’s *Research Ethics* needs a deeper examination of the underlying assumptions and issues in the individual chapters, and also a fuller discussion of the conflicts and complexity of the cases. These topics merit the richer, more extensive consideration of the issues from the perspective of a successful researcher that Kuhar can provide.

## Research ethics and the needs of the community

In the early 1990s when the National Institutes of Health (NIH) initially proposed a requirement for training in the responsible conduct and reporting of research, it was not clear what structure, format, or topics would be optimal. As various institutions developed programs to implement the proposal, review by the NIH identified key elements of effective programs including that these programs should be required, interactive in nature, recurring, and have broad-based faculty involvement^2^ [1, 2]. These characteristics express and exemplify fundamental principles. For example, no matter the discipline, elements of education that are recognized as critical and foundational are not optional, they are required. In addition, interactive discussion (especially when based on true-to-life cases) rather than a purely didactic lecture format is more effective and engaging as a teaching approach/technique [3]. Further, broad-based faculty involvement in RCR education demonstrates that senior researchers (the very individuals who trainees are hoping to emulate) value, and therefore are willing to invest their time in addressing, the responsible conduct of research.

Moreover, as Kuhar correctly points out, training in research ethics is not a “one-and-done” process. Recurring discussion of topics in research ethics is important because, over time, regulations aimed at addressing ethical concerns evolve. More importantly, with experience, the complexity of ethical issues, and the dynamic relationship between different elements and different actors, become increasingly apparent. As a result, trainees often begin to recognize interacting elements in research practice and a broader range of ethical issues. Evolving regulations and researchers’ experience in the context of the research environment, in addition to increasing appreciation of the societal impacts and implications of technology and research findings, mean that continuing education in research ethics is essential. Thus, *Research Ethics in the Life Sciences* both exemplifies and emphasizes early foundational principles of education in the responsible conduct of research.

Practically speaking, *Research Ethics in the Life Sciences* is a paper mentor. Future editions, and their readers, will benefit from both an in-depth, clearer and more nuanced presentation of the ethical and social issues relevant to the various topics, and a more substantial discussion of the cases.

## Footnotes

1 Within the research ethics community, these terms are not always used interchangeably.

2 They should also be embedded in the multiple facets of graduate research training (from regular laboratory meetings to departmental seminars), and include appropriate topics.

## References

[1] Glowinski I. How do you measure success? Benchmarks to guide evaluation of curriculum and instruction. Paper presented at: in Educating for the Responsible Conduct of Research: NIH Policy and Other Mandates, a conference organized by Public Responsibility in Medicine and Research (PRIM&R), Boston (1993).

[2] Bird SJ. Including ethics in graduate education in scientific research. In: Braxton JM, editor. Perspectives on scholarly misconduct in the sciences. Columbus, OH: Ohio State University Press (1999). p. 174–88.

[3] Bransford JD Brown AL Cocking RR. How people learn: brain, mind, experience, and school. Washington D.C.: National Academy Press (2000).

